# The Electrospun Ceramic Hollow Nanofibers

**DOI:** 10.3390/nano7110383

**Published:** 2017-11-09

**Authors:** Shahin Homaeigohar, Yalda Davoudpour, Youssef Habibi, Mady Elbahri

**Affiliations:** 1 Nanochemistry and Nanoengineering, School of Chemical Engineering, Department of Chemistry and Materials Science, Aalto University, Kemistintie 1, 00076 Aalto, Finland; 2 The Institute of Mineralogy, Crystallography and Material Science, Faculty of Chemistry and Mineralogy, University of Leipzig, 04109 Leipzig, Germany; y.davoudpour@gmail.com; 3 Department of Materials Research and Technology (MRT), Luxembourg Institute of Science and Technology (LIST), L-4362 Esch-sur-Alzette, Luxembourg; youssef.habibi@list.lu; 4 Nanochemistry and Nanoengineering, Institute for Materials Science, Faculty of Engineering, Christian-Albrechts-Universität zu Kiel, Kaiserstrasse 2, 24143 Kiel, Germany

**Keywords:** core-sheath nanofibers, hollow nanofibers, electrospinning, ceramic

## Abstract

Hollow nanofibers are largely gaining interest from the scientific community for diverse applications in the fields of sensing, energy, health, and environment. The main reasons are: their extensive surface area that increases the possibilities of engineering, their larger accessible active area, their porosity, and their sensitivity. In particular, semiconductor ceramic hollow nanofibers show greater space charge modulation depth, higher electronic transport properties, and shorter ion or electron diffusion length (e.g., for an enhanced charging–discharging rate). In this review, we discuss and introduce the latest developments of ceramic hollow nanofiber materials in terms of synthesis approaches. Particularly, electrospinning derivatives will be highlighted. The electrospun ceramic hollow nanofibers will be reviewed with respect to their most widely studied components, i.e., metal oxides. These nanostructures have been mainly suggested for energy and environmental remediation. Despite the various advantages of such one dimensional (1D) nanostructures, their fabrication strategies need to be improved to increase their practical use. The domain of nanofabrication is still advancing, and its predictable shortcomings and bottlenecks must be identified and addressed. Inconsistency of the hollow nanostructure with regard to their composition and dimensions could be one of such challenges. Moreover, their poor scalability hinders their wide applicability for commercialization and industrial use.

## 1. Introduction

Nowadays, one-dimensional (1D) nanostructures, including nanofibers, nanotubes, nanorods, nanowires, and nanobelts have drawn immense attention from various scientific communities. This attraction stems from their unique properties, such as size effects, surface effects, and superparamagnetism, leading to their intriguing applications in many advanced areas including sensors, solar cells, nanoresonators, etc. [1]. Among these 1D nanostructures, nanofibers, with large length/diameter ratios, extensive surface area per unit mass, and small diameters (tens to hundreds of nanometers) have been widely spotlighted. The mentioned structural features bring about flexibility in their applicability.

Up to now, techniques such as drawing, template synthesis, phase separation, self-assembly, and electrospinning have been employed for the fabrication of nanofibers made of polymers, metals, ceramics, glass, etc. These methods not only produce nanofibers, but also further assemble them into two-dimensional (2D) and three-dimensional (3D) nanostructures for practical applications [1].

For the last two decades, an almost old technique, called electrospinning, has been underlined mainly because of its extraordinary capabilities in the production of polymer nanofibers [2,3,4]. In this regard, basic electrospinning theories [5,6] have been thoroughly studied, and a diverse range of electrospun nanofibers from various polymer (synthetic or natural) systems, such as neat, nanohybrid and biohybrid, have been made [7,8,9,10,11,12,13,14]. Electrospinning is, in fact, a novel production technique of continuous ultrafine fibers (with diameters from 10 μm to 10 nm) based on forcing a polymer melt or solution through a spinneret with an electrical driving force [15,16]. The produced mats possess small fiber diameters (as mentioned above), highly specific surface areas (tens to hundreds m^2^/g), high porosity, and small pore sizes. Thus, they could be excellent candidates for a wide range of applications, including tissue engineering, drug delivery, textiles, filtration, composite reinforcements, etc. [15,17].

The main advantages of electrospinnig are: its relatively easiness (easy to set up), high speed, low cost, high versatility allowing control over the fibers’ diameters, microstructures and arrangements, and the possibility of a vast selection of materials [15,18,19]. The latter advantage, that is the possibility of fabrication of various nanofiber morphologies and arrangements, is of utmost importance for various applications. By electrospinning, polymers and hybrids thereof can be processed into 1D nanofibers with adjustable compositions, diameters, and porosities [20]. One specific instance is the fabrication of 1D hollow or tubular nanofibers made of ceramics, and, more specifically, of metal oxides, which have been explored extensively for potential applications in catalysis, chemicals, gas sensors, etc. [21,22,23]. 

Hollow nanofibers are able to offer a very extensive surface area that could be highly beneficial for surface-related applications, e.g., chemical sensors, photocatalysis, electromagnetic wave absorbing materials, etc. [24,25]. It is reported that the surface area of the hollow nanofibers is around two times larger than that of the conventional nanofibers [26,27]. In addition to an enhanced surface area, the tubular shape of hollow nanofibers brings about more optimum physicochemical properties for specific applications. For instance, as Choi et al. [28] state, zinc oxide (ZnO) hollow nanofibers show higher electronic transport properties compared to their thin film counterparts. Also, hollow nanofibers are able to provide promising photocatalytic activity [21,29], increased crystallinity [30] and porosity [31,32], optimized electrochemical [33], magnetic, optical and luminescence properties [34,35], as well as high energy storage capacity [36].

In this review, we aim to present a comprehensive overview of the latest development of ceramic hollow nanofiber materials in terms of synthesis approaches, material types, and applications. The emphasis will be on electrospinning for the production of hollow nanofibers, while, with respect to nanofiber materials, we will consider the most common ceramic (metal oxide) materials that have been particularly studied in relevance with energy and environmental remediation applications.

## 2. Electrospinning-Based Fabrication Methods of Ceramic Hollow Nanofibers

The most widely applied process for the fabrication of hollow nanofibers is electrospinning. Unlike the template synthesis and self-assembly methods relying on complex manipulations of molecules to form the desired structure, electrospinning is an efficient and straightforward method to produce hollow nanofibers from either inorganic or organic components [37].

Electrospinning is an effective, adaptable method to form nanofibers with various structures such as, for example, beads on a string, ribbon, cylindrical, grooved, porous, multichannel, core-shell, side by side, helical, hierarchical, and hollow [38,39,40]. Electrospun nanofibers are gaining extensive and growing interest because of their flexibility in terms of size, porosity, surface area, morphology, and surface functionality [41,42].

A simple electrospinning apparatus comprises a Direct Current (DC) high voltage source with positive and negative electrodes connected to a spinning solution container with a nozzle (or spinneret) and a collector, respectively. During the process, an electric field is formed between the spinneret and the collector as a result of their opposite charges. Induced by this electric field, a conical shaped droplet, called “Taylor cone”, is pulled out from the nozzle. Once the electric force dominates the surface tension of the spinning solution, a charged jet is expelled, the solvent gradually evaporates, and the nanofibers are deposited on the collector [43]. Since 2010, various electrospinning procedures and apparatus have been designed for the fabrication of ceramic hollow nanofibers. These approaches include single nozzle, coaxial, microfluidic, triaxial, and emulsion electrospinning (Table 1).

### 2.1. Electrospinning with a Single Spinneret

**Figure 1:**
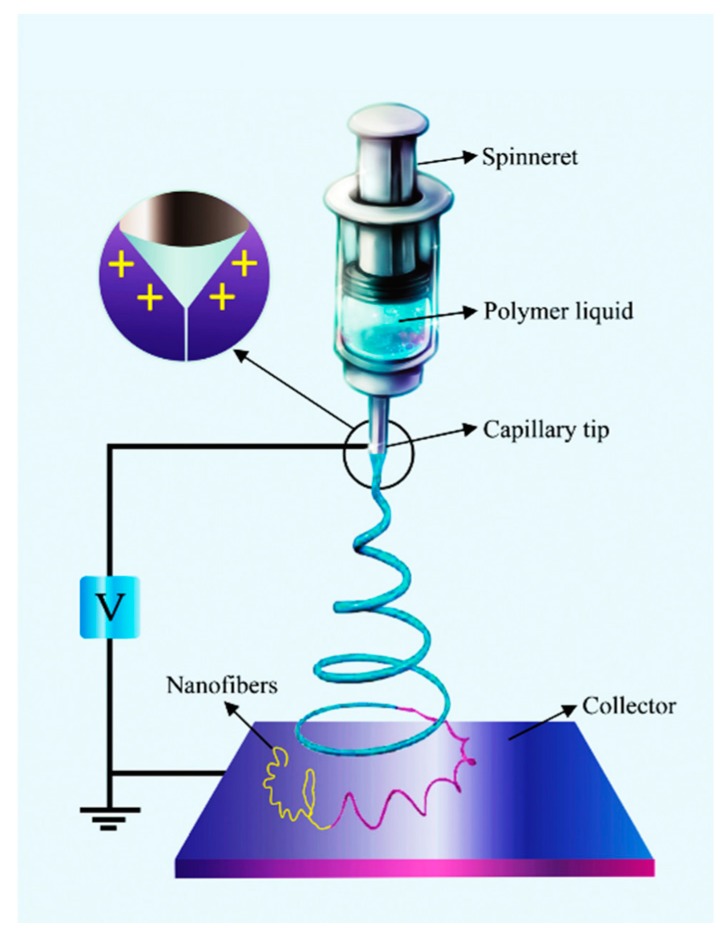
A schematic of the single-spinneret electrospinning (Reproduced with permission from [62]. Royal Society of Chemistry, 2017).

The single-spinneret, or single-nozzle, electrospinning is the simplest electrospinning procedure to form hollow nanofibers from either one component or multiple components [44,52,53,54,55]. The schematic of this process is illustrated in Figure 1. In the case of multicomponents electrospinning, polymers with high and low viscosity move to the inner and outer layers, respectively [56]. The rheological parameters, the solubility of the constituents, and the phase separation of the employed blends influence the homogeneity of their respective solutions [57]. The main challenge in this technique is its low throughput, varying between 1 and 5 mL/h depending on the flow rate of 0.1 to 1 g/h (that is based on the fiber weight), and depending on the operating factors as well as on the solution properties [58,59]. Generally, decreasing the amount of solid materials in the spinning solution as well as the flow rate, declines the nanofibers diameter and the throughput [60]. Despite the simplicity of this process in the lab scale, a high production rate is necessary for industrial and commercial purposes. Moreover, there is a limited number of common solvents to prepare a blend polymer solution, and it is also challenging to find optimum electrospinning conditions for different polymers in a blend solution [20,61].

### 2.2. Coaxial Electrospinning with a Two-Capillary Spinneret

**Figure 2:**
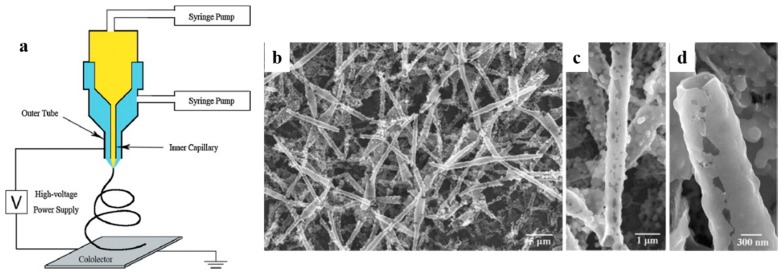
(**a**) A schematic of the coaxial electrospinning process for the fabrication of core-sheath TiO_2_ nanofibers; SEM images of the core-sheath, then hollow TiO_2_ nanofibers (TiO_2_/PVP composite nanofibers were coaxially electrospun with a PVP core solution and a titanium precursor as the shell solution, then calcined at 550 °C for 3 h); (**b**) a low magnification image of the as-synthesized hollow TiO_2_ nanofibrous mat; (**c**) a high magnification image of the TiO_2_ nanofibers; and (**d**) a high magnification image of the cross section of the hollow TiO_2_ nanofiber (Reproduced with permission from [47]. Elsevier, 2017).

Among the derivative techniques of electrospinning, coaxial electrospinning is indeed the most widely employed for the production of hollow nanofibers of polymers [46,63,64], ceramics [47,65], metals [26,45,47,66], and carbon [67,68,69]. In this technique, two different solutions are first fed into a spinneret comprised of two coaxial capillaries, to form a core (inner layer)-sheath (outer layer)-nanofiber structure. Subsequently, by removal of the core via calcination, solvent extraction, or washing, hollow nanofibers are produced [26,46,69,70]. Figure 2a shows schematically the preparation process of core-sheath TiO_2_ nanofibers using a coaxial electrospinning set-up [47]. By removal of the core, hollow TiO_2_ nanofibers are made. Scanning Electron Microscopy (SEM) images of the produced core-sheath, then TiO_2_ hollow nanofibers are shown in Figure 2b–d.

Since the sheath and core solutions meet at the end of the nozzle, two physical phenomena take place simultaneously: the wrapping of the sheath solution around the core solution and the formation of the Taylor cone by the sheath solution to equilibrate the charge effect and the fluid surface tension [71]. Hence, for a successful electrospinning, two important relevant issues should be taken into account. Firstly, the core and the sheath solutions should be immiscible [70,72]. The incompatibility between the core and the sheath solutions governs a gelled interface wherein, by coagulation of both solutions, the hollow nanofibers are produced [73]. Secondly, the sheath solution should be spinnable to impose a shear stress on the core solution while pulling the blended droplet. The core solution can be spinnable or not [47,74]. The flow and diffusion rates, the viscosity, and the miscibility are critical parameters in the coaxial electrospinning process [56]. In contrast to the single-nozzle electrospinning, the key advantage of this method is the possibility of fabrication of hollow nanofibers from a wide variety of materials, even non-electrospinnable solutions [69]. However, Wei et al. [75] have reported the following limitations for this approach:

- The sheath layer must be strong enough to retain the hollow structure, otherwise the produced hollow nanofibers will collapse.
- Despite the easiness of this method, continuous and perfect hollow nanofibers are hardly made because of the post-treatment processes applied to remove the core.
- Complete elimination of the core is challenging.
- The hollow nanofibers prepared by this method can consist of only one layer wall.

Additionally, the limited number of suitable inner solvents and the lack of control over the electrospinning parameters are other problems that can hinder the applicability of the coaxial electrospinning for some systems [76].

### 2.3. Microfluidic Electrospinning

**Figure 3:**
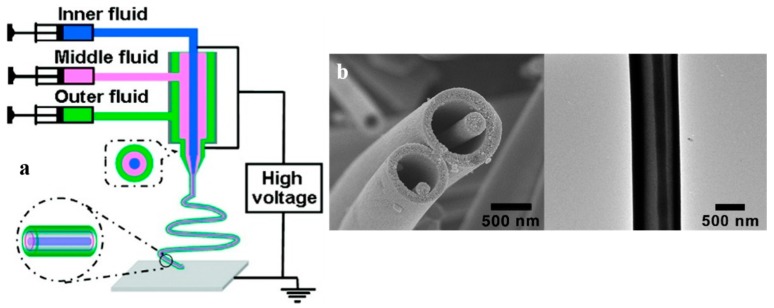
(**a**) The schematic shows the configuration of the microfluidic electrospinning set-up employed to produce hollow TiO_2_ fibers with a nanowire-in-microtube structure. The main spinneret consists of three coaxial capillaries, whereby three fluids are fed to form a compound jet when a high electric field is applied. Among the fluids, the middle one acts as a spacer and separates the inner and outer fluids. (**b**) SEM (left) and TEM (right) images represent the developed nanowire-in-microtube structure (Reproduced with permission from [77]. American Chemical Society, 2017).

If the inner needle of the coaxial electrospinning possesses two or more channels, multichannel hollow nanofibers can be made [48,77,78]. In this procedure, called microfluidic or multifluidic coaxial electrospinning, the apparatus consists of several inner capillaries with an outer nozzle. The outer and inner solutions are separately fed into the capillaries and form a compound Taylor cone that is stretched under an applied electric field and solidified to multichannel nanofibers [48]. Figure 3a illustrates the schematic of a microfluidic electrospinning set-up that is able to fabricate a nanowire-in-microtube structure. This interesting structure is visualized by SEM and Transmission Electron Microscopy (TEM) images (Figure 3b). Compared to the traditional coaxial electrospinning, the microfluidic approach reduces the interaction of the sheath and core fluids, which could be highly miscible or undergo rapid phase separation, by introducing an extra middle fluid as a separator. Thus, a wider range of fluid pairs can be regarded [77]. In addition, the form, size and composition of nanofibers can be properly controlled, as it is desired in the textile and biomedical fields [79]. Other advantages of this process are: simplicity, controllable channel size, rapid prototyping, and parallel spinnability of multiple fibers via arrays of single microchannels [80,81,82]. However, since the inner fluid is surrounded by the middle one, the evaporation of the respective solvents during the process is limited. Thus, this method suffers from a difficulty in the solvent recovery [82].

### 2.4. Triaxial Electrospinning

**Figure 4:**
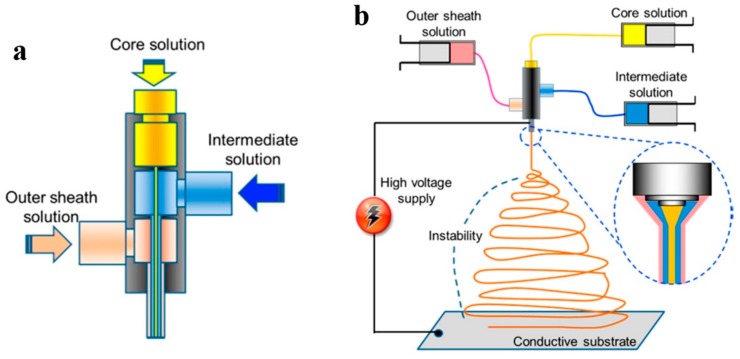
Triaxial electrospinning process: (**a**) triaxial spinneret; (**b**) basic mechanism (Reproduced with permission from [85]. American Chemical Society, 2017).

Triaxial electrospinning employs a spinneret with three concentric needles (Figure 4a). As seen in Figure 4b, three different solutions are pumped into and then meet at the tip of the spinneret. Similar to other electrospinning approaches, the compound solution deforms into a Taylor cone under an electrostatic field. The surface tension of the solution dominates upon the electrostatic force and, thus, a triaxial jet emerges that then experiences bending instability, whipping motion, and solvent evaporation, and eventually it is deposited on the collector as dry fibers [83,84]. This procedure produces three-layered nanofibers including inner (core), intermediate, and outer (sheath) layers. The intermediate layer acts as a barrier between the sheath and the core regions [85].

To successfully perform the triaxial electrospinning, a compound Taylor cone must be formed, and the three involved fluids should be held concentrically together during the procedure [84]. Moreover, it is crucial to select an appropriate solvent for each component, with a boiling point that prevents the solvent from rapidly evaporating, which would damage the structure of the formed nanofibers [86]. The boiling point of the outer layer should be lower than that of the inner layer. Also, the molecular weight of the inner layer should be comparable, or even lower than that of the outer layer. 

Triaxial electrospinning has been applied to fabricate hollow nanofibers from a wide variety of materials [87,88]. For instance, Joo et al. [49] fabricated triaxial electrospun fibers with silica as the shell and core layers, and with a self-assembling polymeric material as the intermediate layer. Also, Chen et al. [77] produced nanowire-in-microtube structured nanofibers through triaxial electrospinning.

The main advantage of this technique is the possibility of formation of nanofibers with a higher surface area enabling the sustained release of important agents [86]. For instance, multidrug delivery vehicles with various release times that are able to sustainably release drugs and improve the healing process can be produced by triaxial electrospinning [85]. Moreover, triaxial electrospinning enables the production of nanofibers from non-electrospinnable components. However, problems such as needle blocking and configuration complexity of this system are challenging [59,89,90].

### 2.5. Emulsion Electrospinning

Emulsion electrospinning, which is the electrospinning of a blend of two immiscible liquid phases, is principally similar to solution electrospinning, but different in chemistry [91,92]. Through this method, discontinuous core-sheath nanofibers are formed by stretching and collapsing of an emulsion [57,93]. Chemically, two dissimilar polymers are dissolved in a solvent, mixed, and settled to produce an emulsion in which the core and sheath segments form from dispersed drops and the continuous phase, respectively [56,91,94,95]. To maintain the stability of the emulsion before and during the jet formation, an emulsifier is usually used. In addition to the stability of the emulsion, the viscosity of the drop phase should be optimum for deformation [56,94,95].

There are two types of emulsion for the electrospinning process: water in oil (W/O) and oil in water (O/W) [95,96]. In a W/O system, the viscosity of the water phase is lower than that of the oily one. Hence, the tendency of the oily phase to form the sheath as a result of its higher viscosity is larger [56,94]. For an O/W system, the situation is the opposite. 

Emulsion electrospinning does not require complex spinnerets compared to the coaxial electrospinning, and it could simply provide good concentric core-sheath nanofibers [97,98]. Another advantage of this method is its eco-friendliness since it employs water rather than organic solvents, and, because of its large dielectric constant, small nanofibers form quickly [95,99]. However, this method suffers from some difficulties in the preparation of a proper emulsion, the elimination of the core, the removal of the emulsifier, which may raise biocompatibility concerns, and a low continuity of the formed hollow nanofibers [52,93].

**Figure 5:**
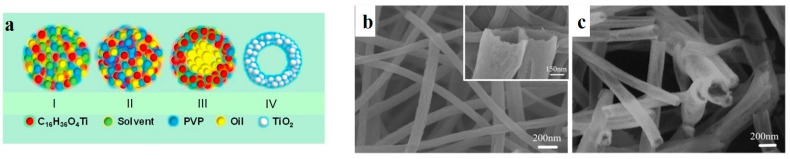
(**a**) The schematic illustrates the formation mechanism of TiO_2_ nanotubes by emulsion electrospinning; SEM images of (**b**) neat TiO_2_ nanotubes (the inset image verifies the nanotubular morphology and rough surface of the formed nanotubes) and (**c**) Ag/TiO_2_ nanotubes (1.5%) (Reproduced with permission from [51]. Elsevier, 2017).

Among the studies on the preparation of core-sheath nanofibers through emulsion electrospinning, Wang et al. [51] made TiO_2_ nanotubes via emulsion electrospinning of a W/O system. As shown in Figure 5a (I), they electrospun a homogenous solution containing PVP and a TiO_2_ precursor (tetrabutyl titanate), wherein a mechanical pump oil was dispersed. During the electrospinning, as shown in Figure 5a (II), the solvent immediately evaporated, leading to the formation of PVP/tetrabutyl titanate nanofibers. The nanofibers also contained oil drops that were insoluble in the precursor solution. As seen in Figure 5a (III), upon drying the electrospun nanofibers at 60 °C for 6 h, the dispersed microdrops of oils coalesce and form larger oil phases. Eventually, when the nanofibers are annealed at 500 °C for 2 h, the oil readily evaporates, and TiO_2_ nanotubes are created (Figure 5a (IV)).

## 3. Most Studied Ceramic Hollow Nanofibers and Their Applications

With respect to hollow nanofiber materials, ceramic—particularly metal oxide—hollow nanofibers have attracted a wide research interest because of their special morphologies, compositions, and chemical and physical properties (e.g., adsorptivity, conductivity etc.) [100]. More specifically, they confer unique electrical, electrochemical, and catalytic properties that are associated with their high surface/volume ratio. Also, in some instances, they offer extraordinary transport properties induced by confinement effects, 1D transport phenomena, or the transport in fractal dimensions [28]. These unique, optimized properties have motivated researchers to employ metal oxide hollow nanofibers as ideal building blocks for a wide range of applications. For instance, they have been evaluated as conductive electrodes for optoelectronic devices (e.g., solar cells [101]), dye adsorbents [102], gas sensors [103,104], chemical sensors [28], etc. In the following table (Table 2), a list of the metal oxide hollow nanofibers developed since 2010 is presented. Afterwards, the most well-known examples of metal oxide hollow nanofibers with their respective applications will be introduced.

### 3.1. Titanium Dioxide (TiO_2_) Hollow Nanofibers for Photodecomposition of Organic Pollutants

TiO_2_ is a semiconducting material with promising characteristics, such as a long-standing stability against chemical and photo corrosion. It is environmentally friendly and shows great photo-reactivity, robust oxidizing activity, and good optical transparency. Moreover, optimum dielectric properties and electrical conductivity, a large refractive index of about 2.52 for anatase and 2.49 for rutile, as well as a large band gap of 3–3.5 eV are other interesting features of TiO_2_ [131]. Thanks to such unique properties, it has been considered for a wide range of applications including solar cells, environmental protection and cleaning, sensors, photocatalysis, photoelectronics, etc. [9,131,132,133,134].

**Figure 6:**
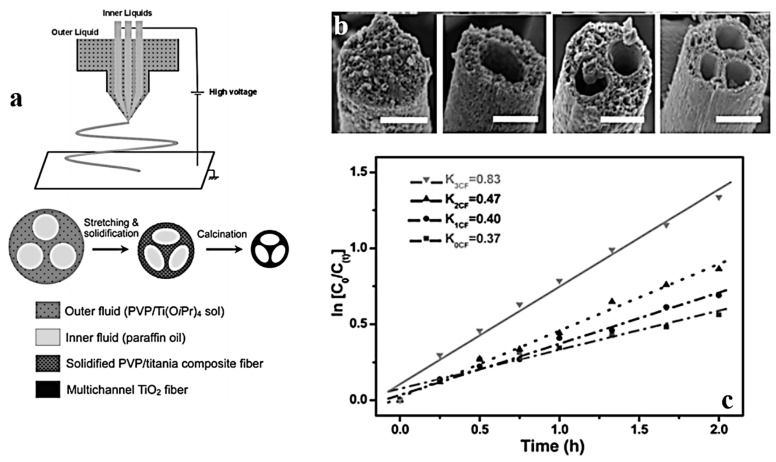
(**a**) The schematic illustration of a multifluidic compound-jet electrospinning method wherein the set-up consists of an outer nozzle and three inner capillaries; (**b**) SEM images of TiO_2_ fibers with 0, 1, 2, and 3 channels (from left to right). The scale bar is 1 µm.; (**c**) The multichannel structure of the hollow TiO_2_ fibers enhances the kinetics of the degradation process of acetaldehyde gas (Reproduced with permission from [48]. Royal Society of Chemistry, 2017).

As a result of the wide band gap, TiO_2_ absorbs Ultraviolet (UV) light and shows optimum photocatalytic activity. This ability is enhanced on large surface areas, e.g., hollow nanostructures, and brings about an efficient degradation of inorganic and organic molecules [135]. In fact, a large surface size leads to a rapid charging–discharging rate because of the small diffusion length and high surface area [136]. In this regard, Zhao et al. [48] introduced nanosized interior hollow channels into TiO_2_ microfibers. The multichannel structured fibers were synthesized by a multifluidic compound-jet electrospinning method (Figure 6a). The optimized photocatalytic activity of the fibers was employed for the degradation of acetaldehyde gas. The authors concluded that the multichannel structure of hollow TiO_2_ fibers (Figure 6b) causes two effects: a multiple reflection of the incident light, and an inner entrapment of the gaseous molecules. As shown in Figure 6c, the photocatalytic degradation of acetaldehyde by TiO_2_ is done via a reaction with first-order kinetics. This fact is confirmed by the linear plot of ln(*C*_0_*/C*_t_) versus the photocatalytic reaction time *t*. While *C*_0_ is the initial concentration of acetaldehyde, *C*_t_ is the concentration of acetaldehyde after the photocatalytic reaction for *t* in hours. This Figure implies that the initial rate constant (*k*) for acetaldehyde degradation increases proportionally to the channel number from solid fibers (*k*_0CF_ ≈ 0.37 h^−1^), one-channel fibers (*k*_1CF_ ≈ 0.40 h^−1^), two-channel fibers (*k*_2CF_ ≈ 0.47 h^−1^), to three-channel fibers (*k*_3CF_ ≈ 0.83 h^−1^). Hollow TiO_2_ nanofibers with a promising photocatalytic effect for the decomposition of the methylene blue (MB) dye have also been synthesized by Chang et al. [47] via coaxial electrospinning. For this, they used titanium sol (a mixture of ethanol, acetic acid, PVP, and titanium butoxide (TBT)) and a titanium precursor (a mixture of TBT and ethylene glycol) for the shell, to produce two types of hollow crystalline TiO_2_ nanofibers. The core fluid was solely a PVP/ethanol/deionized water solution. Both types of nanofibers showed a similar tubular structure. Yet, they were different in terms of surface morphology and shell thickness. When titanium sol was used, a small amount of water in the core hampered the diffusion of the core and shell solutions. Since the titanium precursor is not spinnable, PVP nanofibers were used as core and shell templates during the coaxial electrospinning process. Both hollow TiO_2_ nanofibers were superior to their solid counterparts in terms of MB dye degradation efficiency. In this context, Jung et al. [110] also produced multiwalled carbon nanotube (MWCNT)-embedded TiO_2_ hollow nanofibers that could efficiently photodegrade MB. They stated that the improved degradation efficiency is due to electrons transfer between TiO_2_ and MWCNT and to the MWCNT adsorption ability. The maximum MB decomposition rate of 62% was obtained after 70 min. Regarding another composite hollow nanofiber system, Peng et al. [106] reported that the photocatalytic activity of hollow SnO_2_/TiO_2_ nanofibers prepared by coaxial electrospinning is greater than that of the commercial TiO_2_ photocatalysts. This could be due to their one-dimensional hollow structure and to a continuous hetero-junction between TiO_2_ and SnO_2_. The SnO_2_/TiO_2_ hollow nanofibers could decompose Rhodamine-B (RhB) dye faster than solid TiO_2_ nanofibers and TiO_2_ nanoparticles did. 

In another research, Hou et al. [55] produced mesoporous walled TiO_2_ hollow nanofibers whose composition consisted of rutile (5.4%) and anatase (94.6%). To confer mesoporosity to the nanofibers, a foaming agent was added to the mixed-phase composition. Accordingly, the hollow structure and the mesoporous walls of the nanofibers could cooperatively bring about an excellent photocatalytic efficiency and stability. The as-synthesized nanofibers, whose inner diameter and wall thickness were 215 and 100 nm, respectively, were able to decompose RhB efficiently (99.5%) in 60 min.

**Figure 7:**
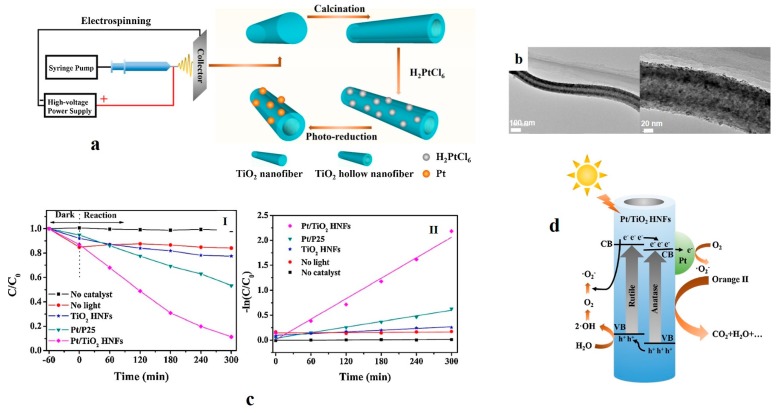
(**a**) The schematic illustration of the entire process of fabrication of Pt/TiO_2_ hollow nanofibers (HNFs); (**b**) TEM image of the 2 wt % Pt/TiO_2_ hollow nanofiber calcined at 350 °C at two magnifications (the scale bars are 100 and 20 nm); (**c**) Photodegradation of Orange II under visible light by various photocatalysts (I) and kinetic graphs relevant to the photodegradation of Orange II (II); (**d**) Schematic illustration of the photodegradation process of Orange II (Reproduced with permission from [112]. Elsevier, 2017).

To shorten the band gap of TiO_2_ in order to extend its photocatalytic applicability to visible light, Yang et al. [112] loaded hollow TiO_2_ nanofibers with Pt nanoparticles. The schematic of the entire process, including electrospinning, calcination, and Pt loading of the nanofibers, is demonstrated in Figure 7a. Moreover, the morphology of the as-synthesized Pt/TiO_2_ hollow nanofiber is depicted in Figure 7b. The nanofibers were made of an anatase–rutile (70:30) mixed phase. By inclusion of Pt (2 wt. %), the band gap of the hollow nanofibers declined from 3.09 to 2.77 eV. This modification resulted in the possibility of a photocatalytic process under visible light. Such a system was studied in terms of degradation of the azo dye orange II. The results represented in Figure 7c imply that such a doped hollow nanofiber is able to degrade the dye molecules with a pseudo-first-rate constant of 0.0069 min^−1^, which was 11.5 and 3.63 times larger than that for the unloaded hollow nanofibers and Pt/P25 (TiO_2_ nanoparticles), respectively. The factors affecting this performance included the Pt loading amount, the calcination temperature of the TiO_2_ hollow nanofibers, the pH of the primary solution, and the light source. The results demonstrated that by addition of 2 wt. % Pt, calcination of the nanofibers at 350 °C, and application of the nanofibers in acidic condition and under solar light, the best photocatalytic activity can be achieved. The main decomposition mechanism of Orange II was attributed to the oxidation by H^+^ and O_2_^−^ radicals, as shown in Figure 7d.

### 3.2. Ferrite Hollow Nanofibers for Electromagnetic and Photocatalytic Devices

Ferrites are natural, abundant, inexpensive, and sustainably permanent magnets. They are known for their spinel shape and the common formula of MFe_2_O_4_, in which M stands for Fe, Co, Ni, or Mn. They show interesting magneto-optical, magnetic, electrical, and magnetoresistive characteristics [137,138]. Such features suggest them as promising candidates for electronic devices (e.g., antenna rod, computer components, and memory devices) [139], batteries, sensors, magnetic recording media, magnetic drug delivery, and catalysis [140]. 

Among various ferrites, the one based on cobalt (Co) has received considerable attention especially for magnetic recording applications because of its anisotropy, optimum chemical stability, mechanical hardness, saturation magnetization, and high coercivity [141,142]. The magnetic properties of this kind of ferrites are influenced by purity, size, and shape of the material, and hence by the manufacturing method [143]. Therefore, it is assumed that a hollow nanostructured ferrite could bring about promising magnetic property. In this regard, Cheng et al. [113] produced CoFe_2_O_4_ hollow nanofibers via electrospinning of a PVP/nitrate salts solution and subsequent calcination. The as-synthesized nanofibers were characterized in terms of crystalline structure, morphology, magnetic properties, etc. The results of X-ray diffraction analysis (XRD), SEM and TEM confirmed the cubic spinel structure, one-dimensional texture, and the existence of many nanoparticles in the wall of the hollow fibers, respectively.

In solution, the carbonyl and tertiary amine groups of PVP can coordinate with Fe^3+^ and Co^2+^, thereby forming a metal–organic framework structure. During the electrospinning process, by evaporation of the solvent (here ethanol/water) the composite fibers are created and can then act as a self-sacrificial template for the CoFe_2_O_4_ hollow nanofibers. Subsequently, the composite fibers are converted to the hollow nanofibers. The mechanism of formation of the hollow nanofibers could be related to the diffusion of the gas product of the PVP decomposition, driving the nanoparticles constituting the fibers from the inside to the outside of the composite fibers. 

As shown in this study, the CoFe_2_O_4_ hollow fibers show a typical ferromagnetic (FM) behavior. As a definition, saturation magnetization (*M*_s_) is the highest induced magnetic moment that a material can get in a magnetic field. As the authors report, the *M*_s_ value of the CoFe_2_O_4_ hollow fibers increases with increasing annealing temperatures. The hollow fibers synthesized in this study are made of many nanoparticles, whose interactions and properties determine the magnetic behavior of the CoFe_2_O_4_ hollow fibers. For small CoFe_2_O_4_ nanoparticles, the inner sides of the nanoparticles are in the usual spin arrangement, whereas the arrangement of their surface atomic moments is disordered. This discrepancy could break their surface exchange bond and change their surface cations coordination, thus decreasing the *M*_s_ of the CoFe_2_O_4_ nanoparticles. As a fact, the higher the calcination temperature is, the larger the particle size will be. Thus, the surface shell contribution to the *M*_s_ would decline when the nanoparticles' size increases. This means that the *M*_s_ would increase at higher annealing temperatures. As shown in this study, at the higher calcination temperature of 700 °C, *M*_s_ for the CoFe_2_O_4_ hollow fibers is 34.71 emu·g^−1^ at 300 K, and 36.82 emu·g^−1^ at 2 K, respectively. This difference can be attributed to the surface spin-canting effect and to the existence of a magnetic dead layer on the surface. When the temperature is low, the surface spins will be frozen along, and thus the local surface anisotropy will be higher. However, at high applied fields, the surface anisotropy once again decreases, and the surface spins are arranged along the field direction, leading to a higher *M*_s_ magnitude. In general, the magnetic measurements verified that the CoFe_2_O_4_ hollow nanofibers are able to offer novel magnetic properties that are promising for electromagnetic and spintronic devices.

**Figure 8:**
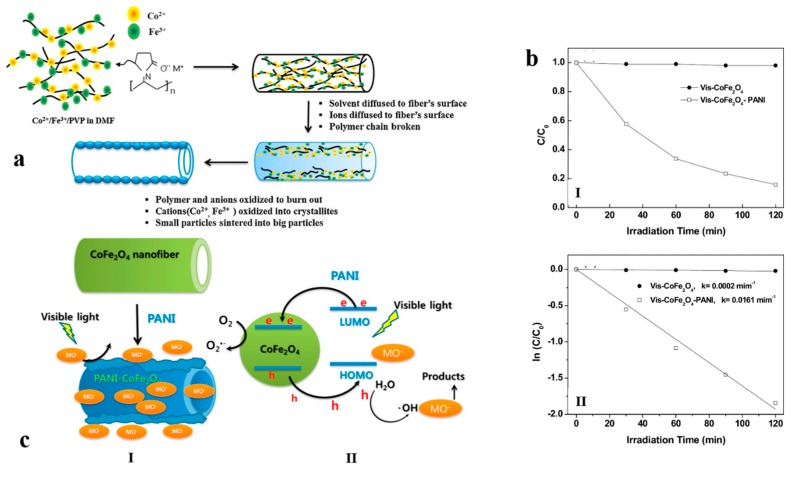
(**a**) Schematic illustration of the formation process of CoFe_2_O_4_ hollow nanofibers; (**b**) (I) visible light photodegradation and (II) kinetic linear simulation curves of the methyl orange (MO) dye for CoFe_2_O_4_ and CoFe_2_O_4_–PANI hollow nanofibers; (**c**) (I) schematic diagram and (II) mechanism of the photodegradation process by CoFe_2_O_4_-PANI hollow nanofiber when subjected to visible light (Reproduced with permission from [115]. Elsevier, 2017).

Other than their electromagnetic properties, CoFe_2_O_4_ hollow nanofibers could also be employed in connection with photocatalytic applications. In this regard, Kim et al. [115] produced hollow core-double-sheath nanofibers with a CoFe_2_O_4_ internal sheath and a PANI external sheath through electrospinning, annealing, and in situ chemical oxidative polymerization. The mechanism of formation of such nanofibers is demonstrated in Figure 8a. The hollow nanofibers were mesoporous, with improved electrical conductivity and optical properties. The hetero-junction made between CoFe_2_O_4_ and PANI enhanced visible light photocatalysis. The authors concluded that the hollow CoFe_2_O_4_-PANI nanofibers harvest visible light, make the quantum confinement impact, assist the electron and mass transfer, and support the charge separation. Thanks to the unique core-shell mesoporous structure of the CoFe_2_O_4_-PANI nanofibers, the pseudo-first-order kinetic constant of the photocatalytic degradation of the methyl orange (MO) dye under visible light was 80 times larger than that for the CoFe_2_O_4_ nanofibers. This finding, along with the photodegradation efficiency of the mentioned nanofibers, is presented in Figure 8b. As shown in Figure 8c, the notably improved photocatalytic activity of the CoFe_2_O_4_-PANI nanofibers is because of the interaction and synergistic effects of CoFe_2_O_4_ and PANI, optimally inducing the separation of electron–hole in the CoFe_2_O_4_ and PANI coupling system.

### 3.3. Iron Compound-Based Hollow Nanofibers for Ferromagnetic Devices

Among the eight known types of iron oxides and different transition metal oxides, magnetite (Fe_3_O_4_), hematite (α-Fe_2_O_3_), and maghemite (γ-Fe_2_O_3_) are widely applied because of their catalytic, biomedical, and magnetic properties. Moreover, their abundance, low-cost processing, lack of toxicity, great theoretical capacity (≈1000 mA·h·g^−1^), and corrosion resistance are promising for a diverse range of applications [144,145,146].

In comparison with other iron oxide polymorphs, α-Fe_2_O_3_ has been widely utilized in batteries, sensors, electrodes, magnetic resonance imaging, transistors, and supercapacitors [147]. To induce a high ferromagnetic property, Cheng et al. [116] synthesized Fe_2_O_3_ hollow nanofibers by calcination of electrospun PVP/Fe(NO_3_)_3_ nanofibers at 500 °C for 4 h, with the heating rate of 1–7 °C·min^−1^. By changing the production conditions, the morphology of Fe_2_O_3_ changes from solid belt to hollow belts and hollow fibers. They found that the key parameter for the hollow nanostructure formation is the PVP decomposition rate, which can be controlled by tuning the heating rate and the rigidity of the surface gel layer. The latter can be changed by the solvent composition as well as by the PVP and Fe(NO_3_)_3_·9H_2_O content. The authors also investigated the magnetic properties of hollow fibers at room temperature, with the field sweeping from −15 to +15 kOe. The hollow fibers remnant magnetization (*M_r_*) and coercivity (*H_c_*) are 0.13 emu·g^−1^ and 177.4 Oe, respectively. These values imply that the hollow fibers show ferromagnetic behaviors at room temperature. Compared to the urchin-like, rod-like, and rhombohedral Fe_2_O_3_ counterparts, the reported values of *M*_r_ and *H*_c_ of the hollow fibers are higher (4.678 × 10^−3^, 2.754 × 10^−3^, and 1.043 × 10^−3^ emu·g^−1^ for *M*_r_, 92.235, 46.94, and 77.75 Oe for *H*_c_, respectively). The reason should be sought in the sensitivity of the magnetization of the ferromagnetic materials to the morphology and structure of the samples made thereof. Here, the assembly of the nanoparticles into the hollow fibers can convert a single domain to a multidomain, thus increasing the *M*_r_ and *H*_c_ values.

**Figure 9:**
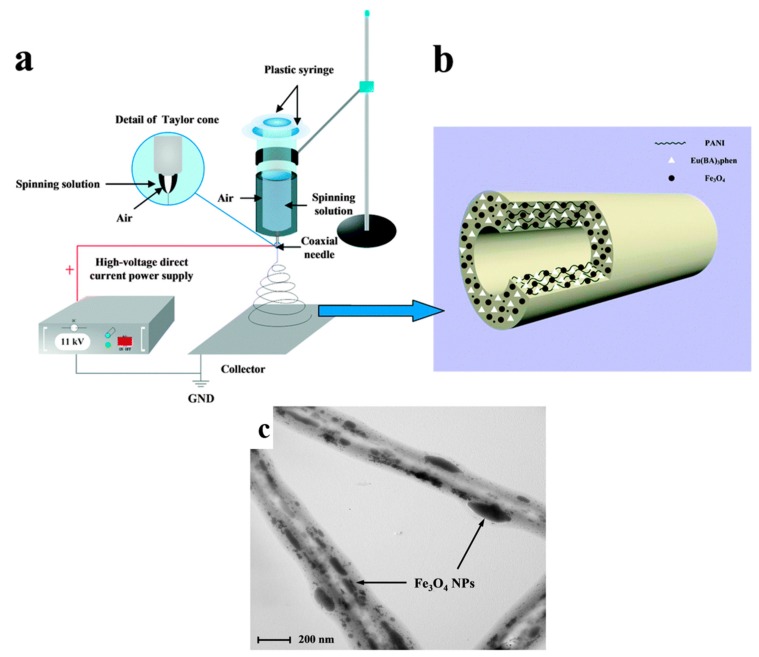
(**a**) The schematic shows details of the one-pot coaxial electrospinning process and set-up; (**b**) the schematic of the as-synthesized hollow nanofibers containing the europium complex, PANI, and Fe_3_O_4_ nanoparticles; (**c**) TEM image of the Eu(BA)_3_phen/PANI/Fe_3_O_4_/PVP hollow nanofibers (Reproduced with permission from [148]. Royal Society of Chemistry, 2017).

Fe_3_O_4_ shows the strongest magnetism among the transition metal oxides. Also, it offers optimum biocompatibility and low level of cytotoxicity in living cells [144]. The preparation of multifunctional hollow nanofibers including a Fe_3_O_4_ component via one-pot coaxial electrospinning without a core solution (using air only), as shown in Figure 9a, has been frequently studied [45,46,148]. For instance, Yu et al. [45] fabricated bifunctional, magnetic luminescent Fe_3_O_4_/Eu(BA)_3_phen/PVP hollow nanofibers. In another work, Liu et al. [148] constructed trifunctional luminescent–electrical–magnetic Eu(BA)_3_phen/PANI/Fe_3_O_4_/PVP hollow nanofibers (Figure 9b,c). The electrical conductivity of 10^−3^ S·cm^−1^ was reported for these hollow nanofibers. The fluorescence emission peaks of Eu^3+^ were detected in the hollow nanofibers and attributed to the ^5^D_0_ → ^7^F_0_ (580 nm), ^5^D_0_ → ^7^F_1_ (592 nm), and ^5^D_0_ → ^7^F_2_ (616 nm) energy level transitions of Eu^3+^ ions. The latter hypersensitive transition induces the strongest emission peak. Interestingly, the luminescent intensity, electrical conductivity, and magnetic properties of the hollow nanofibers were proportional to the amount of Eu(BA)_3_phen, PANI and Fe_3_O_4_ nanoparticles, respectively, and could be adjusted. The photoluminescent–electrical–magnetic trifunctional flexible hollow nanofibers can be proposed for diverse applications, such as electromagnetic interference shielding, microwave absorption, molecular electronics, and biomedicine. 

### 3.4. Zinc Oxide Hollow Nanofibers for Gas Sensing

**Figure 10:**
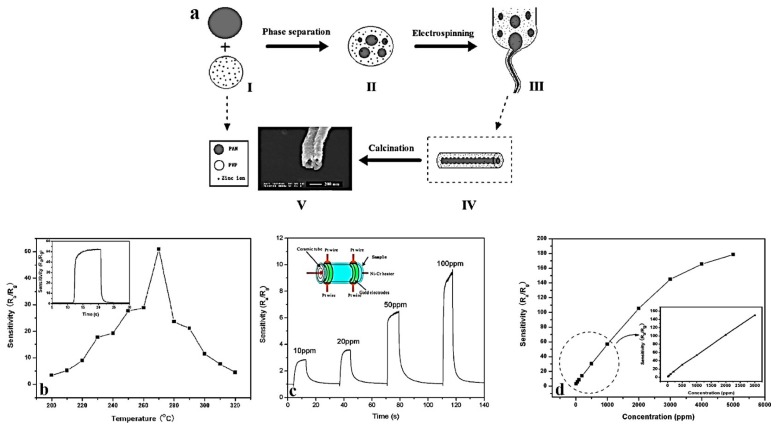
(**a**) The schematic demonstration of various steps of the formation process of ZnO hollow nanofibers; (**b**) ethanol sensitivity of the ZnO hollow nanofibers at different operating temperatures (the inset shows the response–recovery curve of the nanofibers exposed to 1000 ppm of ethanol); (**c**) dynamic response of the sensor to ethanol, whose concentration varies from 10 to 100 ppm (the inset image shows, schematically, the ZnO hollow nanofibers sensor connected to the electrodes); (**d**) the sensor sensitivity versus ethanol concentration (the inset graph implies a linear relationship between sensitivity and ethanol concentration) (Reproduced with permission from [20]. American Chemical Society, 2017).

Zinc oxide (ZnO) is a semiconductor with the band gap energy of 3.37 eV, a large exciton binding energy of 60 meV, excellent chemical and thermal stabilities, and high transparency. Such features enable its use for a wide variety of applications, such as solar cells, sensors, photodetectors, transistors, etc. [149,150,151]. Additionally, ZnO provides a high adsorption capacity for the removal of heavy metals [152], as well as a long life span and great ultraviolet absorption [153]. Electrospun ZnO nanofibers exhibit notable optoelectronic, catalytic, humidity sensing, and piezoelectric properties, as well as a great sensitivity to different gases (e.g., NO_2_, H_2_, CO, C_2_H_5_OH, H_2_S, and NH_3_) [154]. A much larger surface area induced by a hollow structure can notably enhance such promising features. For example, Zhang et al. [20] developed ZnO hollow nanofibers via single-spinneret electrospinning of a precursor solution composed of PAN, PVP, and zinc acetate. The composite nanofibers subsequently underwent thermal decomposition to eliminate the polymers and to fabricate ZnO hollow nanofibers. This process is schematically shown in Figure 10a. During the electrospinning process, a phase separation occurs so that the precursor nanofibers of PAN, PVP, and zinc acetate composite show a core–shell structure consisting of a PAN core and a PVP/zinc acetate composite shell. The as-synthesized ZnO hollow nanofibers can offer remarkable sensing properties against ethanol because of their special one-dimensional hollow nanostructure with an extensive surface area. The sensitivity (*S*) of the sensor follows the equation *S = R*_a_*/R*_g_, where *R*_a_ and *R*_g_ are the sensor resistance in atmospheric air and in ethanol–air mixed gas, respectively. The response and recovery time are the times spent by the sensor to achieve 90% of the total resistance change in the case of adsorption and desorption, respectively.

In the ethanol sensing process by the ZnO hollow nanofibers, oxygen sorption adversely contributes to the electrical transport properties of the nanofibers. When oxygen is adsorbed by the nanofibers, the ZnO conduction electrons are stolen, and thus conductance declines. In the case of the ZnO hollow nanofibers, reactive oxygen species (O_2_^−^, O^2−^, and O^−^) are adsorbed on the inner and outer surfaces of the hollow nanofibers at high temperatures. While at low temperatures, O_2_^−^ is commonly chemisorbed, at high temperatures, O^−^ and O^2−^ are mainly chemisorbed, and O_2_^−^ vanishes immediately. Thus, the reaction kinematics could be represented as: O_2_ (gas) ↔ O_2_ (absorbed) + e^−^ ↔ O_2_^−^ + e^−^ ↔ 2O^−^.

By exposure of the ZnO hollow nanofibers to ethanol, and thus its reaction with ionic oxygen species, the concentration of the oxygen species decreases, and the electron concentration, and thereby the conductance of the ZnO hollow nanofibers, increases. This fact can be demonstrated by the following reaction: CH_3_CH_2_OH (absorbed) + 6O^−^ (absorbed) → 2CO_2_ + 3H_2_O + 6e^−^. The optimum ethanol sensing ability of the ZnO hollow nanofibers could be attributed to their 1D hollow nanofiber structure (with a large length/diameter ratio from the nanofiber structure, and a high surface/volume ratio for the inner and outer surface of the hollow structure). This interesting nanostructure facilitates fast mass transfer of ethanol molecules to and from the interaction region. In addition, it rises the rate for charge carriers to traverse the barriers induced by molecular recognition along the nanofibers.

Figure 10b shows the sensitivity of the ZnO hollow nanofibers when the operating temperature varies. The ethanol concentration used was 1000 ppm. The highest sensitivity (*R*_a_/*R*_g_ = 51) was measured at the temperature of 270 °C. As shown in the inset image, the rapid response and recovery time of the the ZnO hollow nanofiber sensors were around 3 and 5 s, respectively. Figure 10c implies the dynamic response of the sensor when subjected to 10, 20, 50, and 100 ppm of ethanol at 270 °C. The measured sensitivity values were about 2.8, 3.9, 7.2, and 9.4 *R*_a_/*R*_g_ respectively. The response and recovery time were also around 3 and 5 s, respectively. In addition, as shown in Figure 10d reporting the dependence of the sensor sensitivity to the ethanol concentration, the sensor sensitivity increases rapidly with increasing ethanol concentrations, when they are below 2000 ppm. Beyond this concentration, the rate of the response to the ethanol concentration decreases, implying that the sensor becomes more or less saturated. Eventually, at 5000 ppm ethanol, the sensor sensitivity reaches a plateau and a saturation state. The inset graph, showing a linear dependence curve in the range of 10–1000 ppm, further stresses the high ethanol sensing ability of the ZnO hollow nanofibers.

In addition to ethanol, the gas sensing ability of the hollow ZnO nanofibers for formaldehyde [155] and acetone [44] has also been proved. As a composite, Wei et al. [121] synthesized hollow SnO_2_-ZnO nanofibers via single-spinneret electrospinning. These hollow nanofibers showed optimum stability and remarkable sensitivity against toluene at 190 °C, thanks to their hollow structure and the SnO_2_–ZnO hetero-junction. By themselves, SnO_2_ hollow nanofibers have also been considered for magneto-optoelectronic devices, as will be introduced in the next section.

### 3.5. Tin Oxide Hollow Nanofibers for Magneto-Optoelectronic Devices

Among the metal oxides, SnO_2_ has shown an amazing potential as a host lattice for the dilute magnetic semiconductors (DMSs), thanks to its wide band gap, native oxygen vacancies, and high carrier density [34]. DMSs are vastly demanded in modern device applications because they offer ferromagnetism (FM) and semiconducting properties simultaneously. Possessing room temperature FM and high optical transparency, SnO_2_ is one of the most widely studied materials for magneto-opto-electronic devices, as well. By reducing the size of SnO_2_ to nanoscale, the surface area of the ensuing materials increases, which, for instance, is important to improve their magnetic properties [156]. Moreover, quantum confinement effects are extraordinary in 1D structures, resulting in the unusual but beneficial variation of their electronic properties [34]. One of the extensively applied methods for the preparation of SnO_2_ in 1D nanostructures is electrospinning [157,158,159]. In this regard, Xia et al. [37] developed hollow SnO_2_ nanofibers by single-spinneret electrospinning. In this study, the Kirkendall effect, as a result of the concentration gradient between Sn precursors and SnO_2_, was responsible for the formation of hollow SnO_2_ nanofibers. Moreover, the lattice and surface diffusion were found to be the driving forces for the growth of nanograins on the surface of the SnO_2_ nanofibers.

Doping of SnO_2_ confers new properties to the ensuing hollow nanofibers. For instance, Mohanapriya et al. [123] prepared Mn-doped SnO_2_ hollow electrospun nanofibers. The authors observed a reasonable FM transition at 10 K that was attributed to the precipitated impurity phases. In another relevant work, Mohanapriya et al. [34] reported that by doping Cerium (Ce) in SnO_2_, the optical band gap of SnO_2_ hollow nanofibers declined. This confirms the direct energy transfer between f-electrons of Ce ions and the SnO_2_ conduction or valence band. 

**Figure 11:**
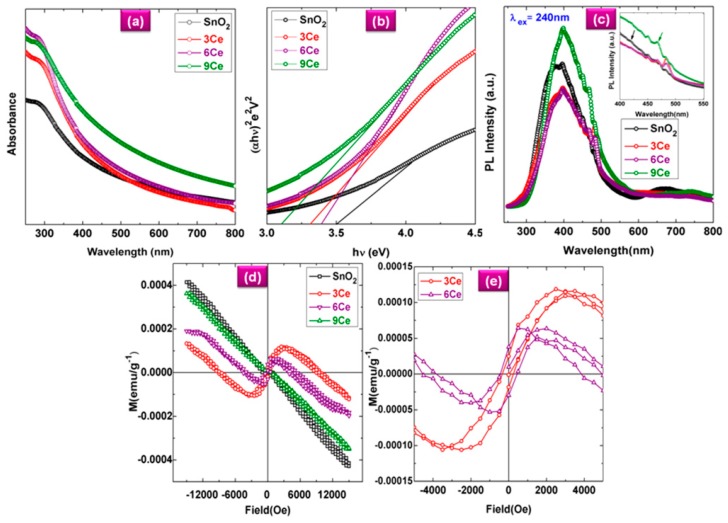
UV-vis absorption spectra (**a**) and Tauc-plot (**b**) of undoped and Ce-doped SnO_2_ hollow nanofibers, (**c**) photoluminescence (PL) spectra of 0, 3, 6, and 9 mol. % Ce-doped SnO_2_ hollow nanofibers, (**d**) M–H curves of 0, 3, 6, and 9 mol. % Ce-doped SnO_2_ hollow nanofibers (at 300 K), and (**e**) extended view of the M–H curve of 3 and 6 mol. % Ce-doped SnO_2_ hollow nanofibers (Reproduced with permission from [34]. AIP Publishing LLC, 2017).

As shown in Figure 11a, the UV absorption edge seen at ~250 nm is correlated with the photo-exciton of charges from the conduction band to the valence band. When the Ce concentration increases, the absorption edge shifts to a higher wavelength corresponding to a smaller crystallite size. This shift is caused by the charge transfer between SnO_2_ valence or the conduction band and the 4f electrons of the Ce ions. The Ce concentration-dependent red shift of the absorption edge witnesses the applicability of the Ce-doped SnO_2_ hollow nanofibers for narrow band-gap optoelectronic devices. The Tauc plots shown in Figure 11b were employed to extract the optical band gap (*E*_g_) for undoped and Ce-doped SnO_2_ hollow nanofibers through the following equation:

(1) $$\alpha hv=A{\left(hv-E_{g}\right)}^{n}$$

where α is the absorption coefficient, *hv* is photon energy, A is constant, *n* = 1/2 for a direct band-gap semiconductor. The *E*_g_ values are obtained via extrapolation at the linear portion of the Tauc plot at α = 0. These values for the undoped and doped SnO_2_ nanofibers are 3.65 and 3.4 eV, respectively. Figure 11c shows the photoluminescence (PL) spectra of the doped and undoped hollow nanofibers. In these graphs, a strong emission band is seen at 240 nm, which is in relevance with the UV excitation. Also, the violet PL emission at 390 nm corresponds to an energy of 3.2 eV, which is lower than that of bulk SnO_2_ (3.6 eV). This discrepancy could be correlated to the direct electronic transition between the donor level and the valence band. As shown in the inset Figure, the Ce-doped SnO_2_ hollow fibers also exhibit an evident blue emission with an additional peak around 420 nm that is induced by presence of the Ce ions. The peaks at 467 nm are attributed to the electron transition between the new unoccupied states of Ce^3+^ 5d, the excited states near the conduction band of SnO_2_, and the 4f states. As demonstrated in Figure 11d,e, the undoped hollow nanofibers can offer only diamagnetism, while the doped ones (containing 3 and 6 mol. % of Ce) show room temperature FM resulting in the FM coupling between s- and f-electrons of the SnO_2_ and Ce ions, respectively. When the doping level reaches 6 mol. %, a room temperature FM property with 19 × 10^−5^ emu·g^−1^ saturation magnetization could be obtained. In general, this study implies the applicability of the Ce-doped SnO_2_ hollow nanofibers for magneto-optoelectronic devices.

### 3.6. Aluminium Oxide Hollow Nanofibers for Dye Adsorption

**Figure 12:**
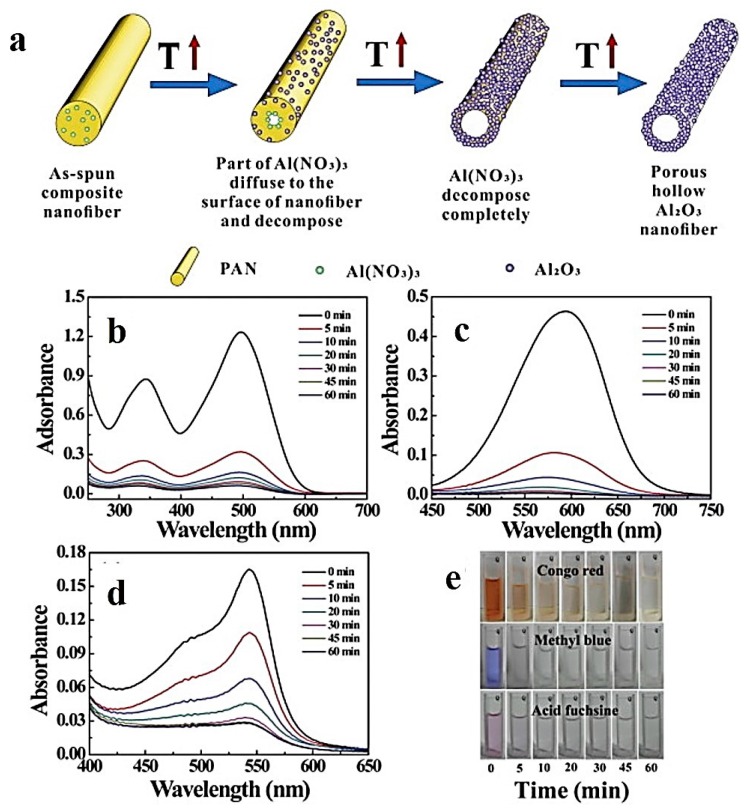
(**a**) The schematic shows different steps of the formation process of the porous hollow γ-Al_2_O_3_ nanofibers; (**b**–**d**) UV–vis spectra of congo red (CR) (**b**) methylene blue (MB) (**c**), and acid fuchsine (AF) (**d**) in proximity of the porous hollow γ-Al_2_O_3_ nanofibers after 0, 5, 10, 20, 30, 45, and 60 min, respectively. (**e**) Camera images of the dye solutions after exposure to the adsorbent at the mentioned time intervals (Reproduced with permission from [124]. Elsevier, 2017).

Alumina (Al_2_O_3_) is a bio-inert ceramic that possesses unique properties, such as high abrasion resistance, biocompatibility, and chemical inertness [160]. Hollow alumina nanofibers prepared via single-spinneret electrospinning and sintering have been studied by Peng et al. [124]. They synthesized γ-Al_2_O_3_ hollow nanofibers, as schematically shown in Figure 12a. The formation mechanism of the hollow nanofibers was based on the Kirkendall effect. The N_2_ adsorption–desorption isotherm was employed to extract the specific surface area of 67.17 m^2^·g^−1^ and the mean pore size of 17.3 nm. The authors proposed that the diameter and pore size of the hollow nanofibers can be controlled through altering the ratio of Al(NO_3_)_3_·9H_2_O to PAN. The hollow nanofibers showed excellent dye adsorption efficiency for three different dyes (i.e., congo red (CR), methylene blue (MB), and acid fuchsine (AF)).

UV-vis adsorption spectroscopy was implemented to determine the dye removal efficiency of the adsorbent. As seen in Figure 12b–d, the notable loss of absorbance proves the high adsorption capacity of the γ-Al_2_O_3_ nanofibers. The camera images shown in Figure 12e further confirm such an ability of the hollow nanofibers versus time. 

In the UV-vis spectra, the absorbance at the corresponding wavelengths of CR, MB, and AF (i.e., 500, 600, and 544 nm, respectively) was the basis to characterize the adsorption performance. As reported by the authors, with respect to CR, the adsorption process proceeds in two stages. First, the adsorption mainly occurred within a short time, so that about 75.28% of CR in the solution was adsorbed in only 5 min. Subsequently, between 5 and 60 min, the adsorption rate decreased. After 60 min, the CR removal efficiency was about 96.52%. The adsorption process for MB proceeded in the same manner. The MB removal efficiency was 72.68% and 92.38% after 5 and 60 min, respectively. On the contrary, for AF, the primary removal after 5 min was not so notable, being only about 32.44%. However, after 60 min, about 91.70% of AF was removed. Thus, in the case of AF, the adsorption was done gradually.

Considering the hydroxylated surface of the γ-Al_2_O_3_ nanofibers, various interactions between γ-Al_2_O_3_ and CR (or MB) could be imagined: (1) likely hydrogen bonding between –OH on the surface of γ-Al_2_O_3_ and the N atom in the dye molecules, (2) electrostatic interactions resulting from excess Al^3+^ ions available on the surface of γ-Al_2_O_3_ nanofibers that can adsorb the dye molecules electrostatically, (3) with a lesser significance, Van Der Waals force that can play a role in the adsorption of the dye onto the surface of the porous adsorbent.

### 3.7. Vanadium Oxide Hollow Nanofibers for Gas Sensing

**Figure 13:**
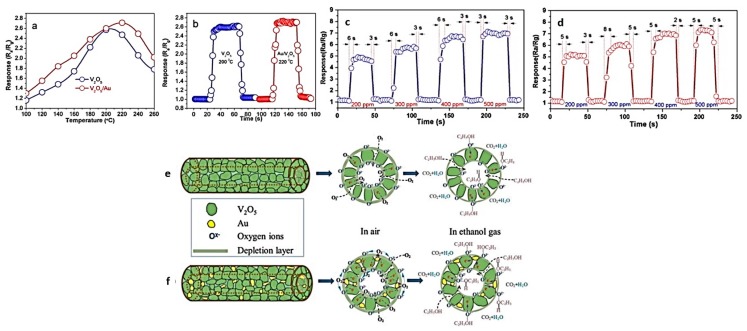
(**a**) Temperature-dependent responses of V_2_O_5_ and Au/V_2_O_5_ nanotubes against ethanol; (**b**) response–recovery behaviors of V_2_O_5_ and Au/V_2_O_5_ nanotubes exposed to ethanol. The dynamic ethanol sensing transients of (**c**) V_2_O_5_ and (**d**) Au/V_2_O_5_ sensors when ethanol concentration changes from 200 to 500 ppm. The optimal operating temperature was regarded for the measurements. The schematic presentation of the sensing mechanism of (**e**) V_2_O_5_ nanotubes and (**f**) Au/V_2_O_5_ nanotubes (Reproduced with permission from [50]. Elsevier, 2017).

Vanadium pentoxide (V_2_O_5_) is a functional ceramic currently stimulating immense research interest for its employment in optical switching devices, catalysis, solar cell, sensors, etc. [10,161]. To maximize the specific surface area, many researchers have attempted to synthesize hollow nanofibers of V_2_O_5_. For instance, Zeng et al. [50] used emulsion electrospinning and then an annealing process to fabricate hollow V_2_O_5_ and Au/V_2_O_5_ nanofibers. Both the hollow nanofibers indicated a quick response–recovery and an outstanding sensitivity against ethanol. As shown in Figure 13a, the hollow nanofibers act optimally at the operating temperatures of 200 and 220 °C, respectively. The Au/V_2_O_5_ show an enhanced performance due to the catalytic effect of Au nanoparticles that improve surface reactions. The response/recovery time of V_2_O_5_ is 5 s/5 s, and the sensing response is ~2.6 s, while such parameters for Au/V_2_O_5_ are 7 and 5 s, and ~2.7, respectively, as shown in Figure 13b. Figure 13c,d, shows the dynamic ethanol sensing transients of two sensors when ethanol concentration varies from 200 to 500 ppm. The response of the sensors depends on the ethanol gas concentration, and a higher sensitivity is always seen for the composite sensor. The sensing mechanism is illustrated in Figure 13e,f, which shows that when the Au/V_2_O_5_ sensor is exposed to air, electrons on the V_2_O_5_ surface move towards the Au nanoparticles mainly because of the Schottky contacts between the Au and the V_2_O_5_ nanoparticles. Concurrently, the embedded Au nanoparticles induce a spill-over effect that contributes to the catalytic activation of oxygen dissociation, enhancing the molecule–ion conversion level, and dissociating oxygen into oxygen species O*^x^*^−^ (O_2_^−^, O^−^ and O^2−^). These species are then transported and distributed onto both the outer and inner surfaces of the nanotubes. As soon as ethanol is released, thanks to the catalytic effect, the sensing reaction between the surface O*^x^*^−^ and the ethanol molecules is facilitated by the Au nanoparticles. This process leads to the transfer of the trapped electrons back to V_2_O_5_, through the following reactions:

O_2_ + 2S_Au_ → 2O-S_Au_ (2)

O-S_M_ + *x*e_vo_^−^ + S_vo_ → O*^x^*^−^ S_vo_ + S_M_ (3)

O*^x^*^−^ S_vo_ + C_2_H_5_OH → 3H_2_O + 2CO_2_ + 6*x*e_vo_^−^ + S_vo_ (4)

where S_Au_ (S_vo_) is an adsorption site on the Au (V_2_O_5_), and e_vo_^−^ is an electron from V_2_O_5_.

## 4. Summary and Remarks on Future Challenges

Hollow nanofibers are a fascinating class of nanomaterials that have gained extensive research interests for advanced applications in energy, environment remediation and biomedicine. This considerable attention stems from their size effects, surface effects, superparamagnetism, large length to diameter ratios, extensive surface area per unit mass, and small diameters. Such amazing features make them eligible for a diverse range of advanced applications in relevance with chemical sensors, photocatalysis, electromagnetic wave absorbing materials, etc.

In this review, we have focused on ceramic hollow nanofibers, and surveyed different synthesis approaches based on electrospinning, as well as their most studied types in terms of composition (i.e., metal oxide types). Among the derivative techniques of electrospinning, coaxial electrospinning is indeed the most widely employed method for the production of ceramic hollow nanofibers. With respect to the ceramic hollow nanofiber material, metal oxides have been extensively investigated for the production of hollow nanofibers for electrodes, photocatalytic adsorbents, sensors etc. For this reason, they were precisely introduced in this review, and their promising features were thoroughly discussed according to the literature. As we mentioned, metal oxide hollow nanofibers are able to develop an extensive surface area that, depending on the employed metal oxide, offers various amazing functionalities. For instance, ZnO and V_2_O_5_ hollow nanofibers provide quick response–recovery and outstanding sensitivity against various gases. As shown for γ-Al_2_O_3_, this feature enables an efficient adsorption-based removal of organics, e.g., dyes, from wastewater streams, electrostatically. In addition, hollow nanofibers of magnetic metal oxides such as SnO_2_, ferrites, and iron oxides induce superior magnetic properties. In addition, the quantum confinement effects are also notable in these 1D structures resulting in the unusual but beneficial variation of their electronic properties. With respect to photocatalytic metal oxides, e.g., TiO_2_, the photocatalytic activity is enhanced in large surface areas of the hollow nanofibers, bringing about the efficient degradation of inorganic and organic molecules. In fact, a large surface area leads to a rapid charging–discharging rate because of the small diffusion length and the high surface area.

Despite highly remarkable properties and potentials for future cutting-edge applications, there are several challenges with regard to the production of ceramic hollow nanofibers. Such difficulties must be addressed and circumvented for the sake of industrialization and upscaling. One of the greatest challenges in this regard is the lack of consistency of the core-shell morphology throughout the length of the fiber. Although what is presented in the literature about the core-shell and hollow fibers often implies a homogenous structure, this is not convincing when a much wider scope, rather than a selected TEM image, is considered. It is noteworthy that a continuous and consistent core-shell morphology necessitates a stable and steady injection of the core and the shell solution during electrospinning. However, this is not possible without any disruption to the core solution feed during the entire spinning process. Also, due to the post-treatment processes applied to remove the core, continuous and perfect hollow nanofibers are hardly made. In fact, the complete elimination of the core is challenging. The sheath layer should be strong enough to retain the hollow structure, otherwise the produced hollow nanofibers will collapse. Additionally, the limited number of suitable inner solvents and the lack of control over electrospinning parameters are other problems that can hinder the applicability of the coaxial electrospinning for some systems. Despite such challenges, the need of a uniform hollow (or core-shell) structure at a large scale depends on the envisaged application. For instance, for the sake of drug delivery, a non-continuous core-shell structure does not affect adversely the drug delivery capability. However, for other applications wherein such a structure is of utmost importance, this requirement should be investigated. As Na et al. [73] suggested, the capillary action of the hollow fiber could be tested to prove the continuity of the hollow fiber structure. For this reason, one end of the hollow fiber is dipped in a fluid, such as silicone oil, while the other end is left open in the air. Under such a circumstance, if the silicone oil permeates throughout the hollow fiber, the fiber is demonstrated to possess a continuous hollow core.

The other significant challenge is related to the scalability of the technique. In general, along with the rapid progress of nanotechnology, nanofibers and their products are more demanded now than ever, but the throughput of the conventional electrospinning is not high enough to satisfy such needs. Therefore, methods able to increase the yields of the electrospun nanofibers are necessary and should be developed.

## Figures and Tables

**Table 1:** Various electrospinning methods for the production of ceramic hollow nanofibers, examples, and detailed operational parameters (studies performed after 2010 were mainly considered).

Method	Precursors	Parameters	Ensuing Hollow Nanofibers	Reference
Single-spinneret electrospinning	Zn(AC)_2_·2H_2_O in water/Poly(vinylpyrrolidon)(PVP) in DMF	Voltage = 17 kV Distance = 20 cm	ZnO	[44]
Coaxial electrospinning	Fe_3_O_4_ nanoparticles/DMF/Chloroform (CHCl_3_)/PVP/Eu(BA)_3_phen powders	Voltage = 11 kV Distance = 12 cm Flow rate = 1 mL·h^−1^	Fe_3_O_4_/Eu(BA)_3_phen/PVP	[45]
Coaxial electrospinning	PVP/Tb_4_O_7_, BA, phen/FeCl_3_·6H_2_O/FeSO_4_·7H_2_O/NH_4_NO_3_, polyethyleneglycol (PEG)/ammonia/oleic acid (OA)/aniline (ANI), (IS)-(+)-camphor-10 sulfonic acid (CSA)/ammonium persulfate (APS)/ethanol (CHCl_3_)/DMF/nitric acid/water	Voltage = 13 Kv Flow rate = 0.0167 mL·min^−1^	Tb(BA)_3_phen/PANI/Fe_3_O_4_/PVP (BA = benzoic acid, phen = phenanthroline, PANI = polyaniline, PVP = polyvinylpyrrolidone)	[46]
Coaxial electrospinning	Titanium butoxide (TBT, Ti(OBu)_4_)/PVP	Voltage = 4–30 kV Distance = 50 cm	TiO_2_	[47]
Microfluidic approach electrospinning	PVP/tetrabutyl titanate (Ti(OC_4_H_9_)_4_/ethanol/paraffin oil	Voltage = 20–30 kV Distance = 15–25 cm Flow rates of inner jet (paraffin oil) = 1.0 mL·h^−^^1^ Flow rates of outer jet (PVP/Ti(OiPr)_4_) = 6–12 mL·h^−^^1^	TiO_2_	[48]
Triaxial electrospinning	tetraethyl orthosilicate (TEOS)/ethanol/water/HCl (shell and innermost layers)+poly(styrene-b-isoprene)(middle layer)	Voltage = 20 kV Distance = 11.5 cm Flow rates of inner jet = 0.02 mL·min^−^^1^ Flow rates of outer jet = 0.03 mL·min^−^^1^	SiO_2_/PS-b-PI/SiO_2_	[49]
Emulsion electrospinning	PVP/VO(acac)_2_/HAuCl_4_·3H_2_O-DMF solution and PS-DMF solution	Voltage = 5–30 kV Distance = 7 cm Flow rate = 2 mL·h^−^^1^	Au/V_2_O_5_	[50]
Emulsion electrospinning	tetrabutyl titanate (C_16_H_36_O_4_Ti)/ethanol/acetic acid + PVP/AgNO_3_/DMF/ethanol + mechanical pump oil	Voltage = 16 kV Distance = 15 cm Flow rate = 2 mL·h^−^^1^	Ag/TiO_2_	[51]

**Table 2:** Various metal oxide hollow nanofibers and their detailed electrospinning parameters.

Hollow Nanofiber	Precursors	Electrospinning Conditions	Annealing Conditions	Reference
TiO_2_	PVP/Tetra-butyl titanate (TBT)/ethanol/acetic acid	*V* = 30 kV *D* = 15 cm	*T* = 500 °C *t* = 4 h HR = 2 °C·min^−1^	[105]
	PVP/tetrabutyl titanate (Ti(OC_4_H_9_)_4_)/ethanol/paraffin oil	*V* = 20–30 kV *D* = 15–25 cm FR (outer) = 6–12 mL·h^−1^ FR (inner) = 1 mL·h^−1^	*T* = 500 °C *t* = 8 h	[48]
	Titanium isopropoxide/PVP/acetic acid/ethanol	*V* = 30 kV *D* = 20 cm FR = 0.1 mL·h^−1^	*T* = 600 °C *t* = 2 h	[66]
SnO_2_/TiO_2_	Titanium isopropoxide/PVP/acetic acid/ethanol	*V* = 5 kV FR (outer) = 1 mL·h^−1^ FR (inner) = 0.1 mL·h^−1^	*T* = 500 °C *t* = 2 h	[106]
TiO_2_	Titaniumisopropoxide(TiP)/poly (methylmethacrylate)(PMMA)/hexadecyl trimethylammoniumbromide/paraffin oil/methylene chloride/ethanol/acetic acid	*V* = 18 kV *D* = 15 cm FR = 100 μL·min^−1^	*T* = 500 °C	[97]
	Titanium butoxide (TBT, I(OBu)_4_)/PVP/ethylene glycol (EG)/ethanol/acetic acid	*V* = 0–50 kV *D* = 50 cm FR = 100 μL·min^−1^	*T* = 550 °C *t* = 3 h HR = 2 °C·min^−1^	[47]
	Titanium (IV) *N*-butoxide (TNBT)/PVP/ethanol/paraffin oil	*V* = 15 kV FR (outer) = 0.8 mL·h^−1^ FR (inner) = 0.6 mL·h^−1^	*T* = 500 °C *t* = 6 h	[107]
	polyacrylonitrile (PAN)/PVP/dimethylformamide (DMF)/tetrabutyl titanate (Ti(OC_4_H_9_)_4_)		*T* = 500 °C *t* = 5 h	[108]
BaTiO_3_	Barium acetate/acetic acid/Titanium (IV)-isopropoxide/PVP/ethanol	*V* = 12 kV *D* = 15 cm FR = 0.3 μL·s^−1^	*T* = 500, 700, 950 °C *t* = 1 h HR = 2.5 °C·min^−1^	[109]
Carbon nanotube (CNT)-TiO_2_	PAN/Multiwalled CNTs (MWCNTs)/DMF/titanium tetra-isopropoxide (TTIP)/isopropyl alcohol	*V* = 18 kV *D* = 10 cm FR = 1 mL·h^−1^	*T* = 550 °C *t* = 1 h	[110]
TiO_2_	Butyl titanate (TBOT)/diiso-propyl azodiformate (DIPA)/paraffin oil/ethyl alcohol/acetic acid/deionized water	*V* = 18 kV *D* = 20 cm FR = 1 mL·h^−1^	*T* = 500 °C *t* = 3 h HR = 1 °C·min^−1^	[55]
	Polyvinyl acetate (PVAc)/titanium isopropoxide (TIP)/DMF/calcium carbonate (CaCO_3_)/hydrochloric acid (HCl)	*V* = 17 kV *D* = 18 cm FR = 1 mL·h^−1^	*T* = 500 °C *t* = 3 h	[111]
Pt/TiO_2_	Tetrabutyl titanate (Ti(OC_4_H_9_)_4_,TBOT)/ethanol/hexachloro-platinic acid (H_2_PtCl_6_·6H_2_O)/PVP/Nitric acid(HNO_3_)	*V* = 25 kV *D* = 25 cm FR = 1.3 ± 0.02 mL·h^−1^	*T* = 350–500 °C *t* = 4 h	[112]
CoFe_2_O_4_	PVP/Fe(NO_3_)_3_·9H_2_O/Co(NO_3_)_2_·6H_2_O/ethanol/water	*V* = 30 kV *D* = 15 cm FR = 1.3 ± 0.02 mL·h^−1^	*T* = 500–600–700 °C *t*= 4 h HR = 3 °C·min^−1^	[113]
CuFe_2_O_4_	PVP/Fe(NO_3_)_3_·9H_2_O/Cu(NO_3_)_2_·3H_2_O/ethanol/water	*V* = 15 kV *D* = 15 cm	*T* = 500 °C *t* = 2 h HR = 0.5 °C·min^−1^	[114]
CoFe_2_O_4_–PANI	Cobalt(II) nitrate hexahydrate (Co(NO_3_)_2_·6H_2_O/iron(III) nitrate enneahydrate (Fe(NO_3_)_3_/ethanol/PVP/ammonium peroxodisulfate	*V* = 20 kV *D* = 17 cm FR = 0.5 mL·h^−1^	*T* = 550 °C *t* = 2 h HR = 5 °C·min^−1^	[115]
SrFe_12_O_19_	Strontium nitrate (Sr(NO_3_)_2_)/Ferric nitrate (Fe(NO_3_)_3_·9H_2_O)/PVP/DMF	*V* = 15 kV *D* = 15 cm FR = 0.5 mL·h^−1^	*T* = 600–650–700–750 °C *t* = 3 h HR = 1 °C·min^−1^	[26]
Fe_2_O_3_	PVP/Fe(NO_3_)_3_·9H_2_O/water/ethanol	*V* = 30 kV *D* = 15 cm	*T* = 500 °C *t* = 4 h HR = 1–7 °C·min^−1^	[116]
MnO_2_-doped Fe_2_O_3_	Citric acid/ferric citrate/deionized water/manganese acetate	*V* = 15 kV *D* = 10 cm	*T* = 400 °C *t* = 4 h HR = 0.5 °C·min^−1^	[117]
Fe_3_O_4_/Eu (BA)_3_phen/PVP	Fe_3_O_4_ nanoparticles/DMF/CHCl_3_/PVP/Eu (BA)_3_phen powders	*V* = 11 kV *D* = 12 cm		[45]
Tb(BA)_3_phen/PANI/Fe_3_O_4_/PVP	Benzoic acid (BA)/phenan-throline (phen)/polyaniline (PANI)/PVP/sulfonic acid/ammonium persulfate/ethanol/CHCl_3_/DMF/nitric acid/deionized water/Tb_4_O_7_	*V* = 13 kV *D* = 14 cm FR = 0.0167 mL·min^−1^		[46]
Carbon-coated LiFePO_4_	Lithium dihydrogen phosphate (LiH_2_PO_4_)/iron nitrate 9-hydrate ((Fe(NO_3_)_3_·9H_2_O)/ferrous sulfate 7-hydrate (FeSO_4_·7H_2_O)/DMF/PMMA	*V* = 16 kV *D* = 15 cm FR (outer) = 0.2 mL·h^−1^ FR (inner) = 0.4 mL·h^−1^	*T* = 750 °C *t* = 3 h HR = 2 °C·min^−1^	[118]
CuO	PVP/copper acetate (Cu(CH_3_COO)_2_)/ethanol	*V* = 10 kV *D* = 13 cm FR = 0.02 mL·min^−1^	*T* = 500 °C *t* = 2 h HR = 6.7 °C·min^−1^	[119]
CuO	Copper (II) sulfate pentahydrate (CuSO_4_·5H_2_O)/PVP/water	*V* = 16.8 kV FR = 6 μL·min^−1^	*T* = 673 and 873 K *t* = 5 h	[120]
SnO_2_-ZnO	Zn(AC)_2_·2H_2_O/SnCl_2_·2H_2_O/PVP/DMF/ethanol/ethyl acetate	*V* = 19 kV *D* = 20 cm FR = 0.7 mL·h^−1^	*T* = 600 °C *t* = 3 h	[121]
SnO_2_	Stannic chloride pentahydrate (SnCl_4_·5H_2_O)/ethanol/DMF/PVP	Electric field = 1.25 kV/cm *D* = 18 cm FR = 0.2 mL·h^−1^	*T* = 550–650°C *t* = 4 h	[122]
Mn-Doped SnO_2_	SnCl_2_·2H_2_O/DMF/ethanol/PVP/Mn(CH_3_COO)_2_·4H_2_O	*V* = 25 kV *D* = 18 cm FR = 1mL·h^−1^	*T* = 600 °C *t* = 3 h	[123]
Cerium-doped SnO_2_	SnCl_2_·2H_2_O/DMF/ethanol/PVP/Ce(NO_3_)_3_·6H_2_O	*V* = 25 kV *D* = 18 cm	*T* = 600 °C *t* = 5 h HR = 5 °C·min^−1^	[34]
Al_2_O_3_	Aluminum nitrate (Al(NO_3_)_3_)/PAN/DMF	*V* = 20 kV *D* = 20 cm FR = 1 mL·h^−1^	*T* = 500–1000–1300 °C HR = 5 °C·min^−1^	[54]
γ-Al_2_O_3_	Aluminum nitrate (Al(NO_3_)_3_)/PAN/DMF	*V* = 20 kV *D* = 20 cm FR = 1 mL·h^−1^	*T* = 800 °C *t* = 2 h HR = 5 °C·min^−1^	[124]
Au/V_2_O_5_	Vanadyl acetylacetonate (VO(acac)_2_)/gold(III) chloride trihydrate (HAuCl_4_·3H_2_O)/PVP/polystyrene (PS)	*V* = 20 kV *D* = 20 cm FR = 2 mL·h^−1^	*T*_1_ = 330 °C *t*_1_ = 2 h HR_1_ =5 °C·min^−1^ *T*_2_ = 330–430 °C *t*_2_ = 30 min HR_2_ = 2°C·min^−1^	[50]
Vanadium nitride (VN)	Oxalic acid dihydrate (C_2_H_2_O_4_·2H_2_O)/ethanol/PVP/ammonium metavanadate (NH_4_VO_3_)	*V* = 15 kV *D* = 15 cm	*T* = 400–600–800 °C *t* = 1 h HR = 2 °C·min^−1^	[125]
CNTs/InVO_4_	Multi-walled carbon nanotubes/In(NO_3_)_3_·4.5H_2_O/C_10_H_14_O_5_V/PVP/ethanol	*V* = 21 kV *D* = 15 cm	*T* = 550 °C *t* = 2 h	[126]
Te	Ni acetate/PVP/HTeO^2+^			[127]
LiFePO_4_/C/Ag	Fe(NO_3_)_3_·9H_2_O/AgNO_3_/H_3_PO_4_/LiOH·H_2_O/DMF/PVP	*V* = 13 kV *D* = 16 cm	*T* = 700 °C *t* = 10 h HR = 1 °C·min^−1^	[128]
Chromium-doped spinel	Zn(NO_3_)_2_·6H_2_O/Mg(NO_3_)_2_·6H_2_O/Al(NO_3_)_3_·9H_2_O/Cr(NO_3_)_3_·9H_2_O/ethanol/deionized water/PVP	*V* = 20 kV *D* = 12 cm FR = 1.5 mL·h^−1^	*T* = 1000–1100–1200 °C *t* = 5 h HR = 200 °C·h^−1^	[129]
YF_3_:Eu^3+^	Yttrium oxide (Y_2_O_3_)/europium oxide (Eu_2_O_3_)/DMF/ammonium hydrogen fluoride (NH_4_HF_2_)/Nitric acid (HNO_3_)/ethyl alcohol	*V* = 13 kV *D* = 16 cm	*T*_1_ = 700 °C *t*_1_ = 8 h HR_1_ = 1 °C·min^−1^ *T*_2_ = 200 °C HR_2_ = 1 °C·min^−1^	[130]
YF_3_:Yb^3+^/Er^3+^	Yttrium oxide (Y_2_O_3_)/erbium oxide (Er_2_O_3_)/PVP/DMF/ammonium hydrogen fluoride (NH_4_HF_2_)/Nitric acid (HNO_3_)/	*V* = 16 kV *D* = 18 cm	*T*_1_ = 700 °C *t*_1_ = 8 h HR_1_ = 1 °C·min^−1^ *T*_2_ = 200 °C HR_2_ = 1 °C·min^−1^	[35]

*V* = voltage, *D* = distance between nozzle to collector, FR = flow rate, *T* = temperature, *t* = time, HR = heating rate.
